# Evaluation of Hypertension in Pediatric Patients With Systemic Lupus Erythematosus: A Historical Study

**DOI:** 10.1155/ijhy/1352079

**Published:** 2026-08-29

**Authors:** Mohamed Husein Aldokhi, Noosha Samieefar, Fatemeh Tahghighi, Behnaz Bazargani, Arash Abbasi, Daryoush Fahimi, Fahimeh Askarian, Mastaneh Moghtaderi

**Affiliations:** ^1^ International College of Tehran, University of Medical Science, Tehran, Iran, mubabol.ac.ir; ^2^ Pediatric Chronic Kidney Disease Research Center, Gene, Cell & Tissue Research Institute, Children’s Medical Center, Tehran University of Medical Sciences, Tehran, Iran, tums.ac.ir; ^3^ Network of Interdisciplinarity in Neonates and Infants (NINI), Universal Scientific Education and Research Network (USERN), Tehran, Iran, usern.tums.ac.ir; ^4^ Pediatric Rheumatology, Pediatric Rheumatology Research Group, Rheumatology Research Center, Tehran University of Medical Sciences, Tehran, Iran, tums.ac.ir

**Keywords:** hypertension, kidney diseases, lupus erythematosus, pediatrics systemic

## Abstract

**Introduction:**

Childhood‐onset systemic lupus erythematosus (cSLE) primarily affects children and adolescents and is a potentially life‐threatening condition. In contrast to adult‐onset SLE, children with cSLE tend to have a more severe and aggressive course of disease. One of the most prevalent and concerning comorbidities in SLE patients is hypertension (HTN). This study evaluates diverse aspects of HTN in pediatric patients afflicted with cSLE at a tertiary hospital.

**Method:**

This historical study includes medical records of all patients with cSLE referred to Children’s Medical Hospital for two years (2020–2022). Data were collected on various clinical features and laboratory findings and were analyzed by SPSS software.

**Results:**

A total of 76 patients with cSLE were included, of whom 59.2% had lupus nephritis at diagnosis. Forty‐one patients (53.9%) had normal blood pressure, while 35 patients (46.1%) were diagnosed with HTN. Although the mean age of the HTN group was higher than that of the non‐HTN group, the difference, however, was not statistically significant (*p* value = 0.548). Similarly, the gender distribution between the HTN and non‐HTN groups was not significantly different (*p* value = 0.350), suggesting that neither age nor sex is a major determinant of HTN in this population. Significant differences were observed between the HTN and non‐HTN groups in hemoglobin, hematocrit, polymorphonuclear cell count, complement Component 3, creatinine, blood urea nitrogen, sodium, and calcium levels.

**Conclusion:**

Laboratory changes associated with increased disease activity, including lower C3 levels, lower hemoglobin, and higher BUN and Cr were reported in cSLE patients with HTN. Given the high prevalence of HTN among cSLE patients, early diagnosis and treatment of HTN and renal involvement are crucial in the long‐term outcomes of these patients.

**Key Clinical Message**
• The disease course of SLE is more severe in pediatric patients, and early diagnosis and treatment of hypertension (HTN) and renal involvement are crucial for improving long‐term outcomes. Laboratory changes associated with increased disease activity were reported in cSLE patients with HTN. Given the high prevalence of HTN in cSLE, regular monitoring and a tailored approach to management are essential to improve patient outcomes and reduce long‐term damage in this complex multisystem disease.

## 1. Introduction

Childhood‐onset systemic lupus erythematosus (cSLE), affecting children under 18 years of age, is a multisystem autoimmune disease resulting from immune dysregulation. This disease is known for its complexity and wide range of clinical features, which make its diagnosis challenging for healthcare professionals. Well‐established symptoms of cSLE include fever, fatigue, weight loss, joint pain, skin rash, and renal involvement. In comparison to adults, the childhood type has a more severe and aggressive course [1–3]. At presentation and during the disease course, cSLE patients have more active disease, particularly renal involvement. Children also receive more intensive medication and experience more drug‐induced side effects, mostly related to steroid toxicity [4].

Lupus nephritis (LN) encompasses a variety of patterns of inflammatory renal involvement, including glomerular and tubulointerstitial pathology [5]. The diagnosis should meet the American College of Rheumatology (ACR) criteria: persistent proteinuria > 0.5 gr/day or higher than +3 by dipstick and/or the presence of cellular casts including red cell, hemoglobin (Hgb), granular, tubular, or mixed in the urine. A revision of the ACR criteria recommended substituting 24‐h protein quantification with a spot urine protein‐to‐creatinine ratio of more than 0.5 which is considered the main predictor of a poor renal prognosis in SLE [6].

HTN, a prevalent condition in cSLE, is a major risk factor for the development and progression of cardiovascular and renal complications [7]. Recent studies have revealed HTN in cSLE to be associated with obesity and renal dysfunction [7, 8]. While the exact pathophysiology of HTN in SLE patients is not fully understood, studies have suggested a combination of factors, such as inflammation, immune system dysfunction, metabolic and hormonal changes, and drug side effects [9–11]. The risk factors for end‐stage renal disease (ESRD) in pediatric LN include Class IV disease, HTN, high levels of creatinine (Cr), and low levels of Component 3 (C3) complement at presentation [11].

Early detection and management of HTN is essential in cSLE patients to help prevent the progression of cardiovascular and renal involvement. Monitoring blood pressure in all cSLE patients, not just those with renal involvement, is crucial [12]. In conclusion, a better understanding of HTN in cSLE patients can help improve their long‐term outcomes and quality of life. This study was designed to evaluate diverse aspects of HTN in pediatric patients afflicted with cSLE at the Children’s Medical Center hospital.

## 2. Methods

This historical study analyzed medical records from the Children’s Medical Center hospital for patients with cSLE from 2020 to 2022. Patients included in the study met the 2019 revised ACR criteria for the diagnosis of SLE and had an age of onset under 18 years. Blood pressure was measured three times in outpatient setting and every 6 h in hospitalized patients to ensure accuracy and consistency for establishing the diagnosis of HTN. Ambulatory blood pressure monitoring is regarded as the gold standard for detecting HTN. However, in this study, blood pressure was measured by an expert physician on three separate visits. Elevated blood pressure was defined according to guideline‐based percentiles adjusted for the patient’s age, sex, and height. Readings above the 90th percentile were classified as elevated, while those at or above the 95th percentile were considered HTN. In other words, a blood pressure at the 95th percentile indicates that 95% of children of the same age, sex, and height have lower values.

The last referral between 2020 and 2022 was considered the final visit. Data of the first visit (disease onset) were extracted from the medical records on various clinical features, including blood pressure measurements, laboratory findings, damage accrual, and mortalities (renal, pulmonary, cardiac, gastrointestinal, and neurologic). Echocardiography was performed in all patients to evaluate potential cardiac involvement. Renal involvement was defined as a rise in Cr or episodes of hematuria and/or proteinuria (LN). Pulmonary involvement was diagnosed by chest X‐ray. Gastrointestinal complications were evaluated by a history of related symptoms.

Laboratory data included measurements of Hgb, hematocrit (HCT), white blood cell (WBC) count, lymphocyte (LYM) cell count, polymorphonuclear (PMN) cell count, platelet (PLT) count, thyroid‐stimulating hormone (TSH), cholesterol (Chol), triglycerides (TG), antinuclear antibody (ANA), complement C3, complement Component 4 (C4), erythrocyte sedimentation rate (ESR), C‐reactive protein (CRP), Cr, blood urea nitrogen (BUN), potassium (K), sodium (Na), phosphorus (Ph), magnesium (Mg), calcium (Ca), PH of venous blood gas (pH‐VBG), partial pressure of oxygen (PO_2_‐VBG), partial pressure of carbon dioxide (PCO_2_‐VBG), and bicarbonate of VBG at both the initial and follow‐up visits. These parameters were used to assess renal function and systemic disease activity over time.

Data analysis was performed using the SPSS software, Version 26 (SPSS Inc., Chicago, USA). Descriptive statistics, including mean, standard deviation (SD), and frequencies, were calculated to summarize quantitative and categorical variables. Comparative analyses between groups (e.g., patients with and without HTN) were performed using the independent *t*‐test for continuous variables and the chi‐square test for categorical variables. We considered *p* values of lower than 0.05 statistically significant.

Ethical approval for this study was obtained from the Ethics Committee of Tehran University of Medical Sciences, allowing access to and extraction of patient data from hospital files and records (Ethics code: IR. TUMS. CHMC. REC. 1402. 05).

## 3. Results

Among 76 patients with cSLE, 56 (73.7%) were female and 20 (26.3%) were male. The mean age at disease onset was 11.061 ± 3.8 years. Mean weight and height were 36.149 ± 16.2 kg and 138.330 ± 24.9 cm, respectively. Kidney biopsy was performed in 45 patients, and histopathology examination revealed Class II LN in 4 patients (8.8%), Class III in 11 patients (24%), Class IV in 28 patients (62.2%), and Class V in 2 patients (4.4%).

The most common presenting symptom was arthralgia, observed in 52.6% of patients. The lowest frequent presentation was the Raynaud phenomenon, presented in only one patient (1.3%). Notably, fever was present in 40.8% (*N* = 31) of patients, while chills were less commonly reported, with a prevalence of 7.9% (*N* = 6). Neurological manifestations were infrequent, with seizures and psychosis occurring in 10.5% (*N* = 8) and 3.9% (*N* = 3) of patients, respectively. Headache was reported by 11.8% of patients (*N* = 9). The distribution of symptoms, signs, and complications is available in Table 1.

**TABLE 1:** The frequency of symptoms, signs, and complications.

Variable	Absence of symptom/sign/complication number (%)	Presence of symptom/sign/complication number (%)
Fever	42 (55.3)	31 (40.8)
Chill	67 (88.2)	6 (7.9)
Arthritis	58 (76.3)	15 (19.7)
Arthralgia	33 (43.4)	40 (52.6)
Malar rash	60 (78.9)	14 (18.4)
Hair loss	65 (85.5)	8 (10.5)
Skin rash	47 (61.8)	26 (34.2)
Raynaud phenomena	72 (94.7)	1 (1.3)
Seizure	65 (85.5)	8 (10.5)
Psychosis	70 (92.1)	3 (3.9)
Headache	64 (84.2)	9 (11.8)
Nephritis	31 (40.8)	45 (59.2)
Serositis	71 (93.4)	2 (2.6)
Mucositis	56 (73.7)	17 (22.4)
Pulmonary involvement	62 (81.6)	11 (14.5)
Cardiac involvement	57 (75.0)	16 (21.1)
GI involvement	53 (69.7)	19 (25.0)

Thirty patients (39.5%) had hematuria at the first visit, which decreased to 25 patients (32.9%) at the last visit. Proteinuria showed temporal variation, with the proportion of patients testing negative increasing from 35.5% (*N* = 27) at the first visit to 51.3% (*N* = 39) at the last visit. Glycosuria remained rare throughout the study. Most patients (86.8%, *N* = 66) at the first visit and 78.9% (*N* = 60) at the last visit were negative for glycosuria, with only minor variations over time. Urinalysis results of patients are provided in Table 2.

**TABLE 2:** Urinary analysis of patients.

Variable	Missing	Negative number (%)	Trace number (%)	One plus number (%)	Two plus number (%)	Three plus number (%)	Four plus number (%)
Hematuria in the first visit	6	40 (52.6)	2 (2.6)	11 (14.5)	8 (10.5)	5 (6.6)	4 (5.3)
Hematuria in the last visit	6	45 (59.2)	1 (1.3)	5 (6.6)	6 (7.9)	6 (7.9)	7 (9.2)
Proteinuria in the first visit	7	27 (35.5)	8 (10.5)	14 (18.4)	9 (11.8)	10 (13.2)	1 (1.3)
Proteinuria in the last visit	7	39 (51.3)	1 (1.3)	10 (13.2)	6 (7.9)	12 (15.8)	1 (1.3)
Glycosuria in the first visit	7	66 (86.8)	—	2 (2.6)	—	1 (1.3)	—
Glycosuria in the last visit	7	60 (78.9)	3 (3.9)	2 (2.6)	2 (2.6)	—	—

In this study, 41 patients (53.9%) had normal blood pressure, while 35 patients (46.1%) were diagnosed with HTN. The mean age of the HTN group was 11.35 ± 3.4 years, which was higher than the mean age of the non‐HTN group (10.81 ± 4.2 years), although this difference was not statistically significant (*p* value = 0.548). In the HTN group, 24 patients (68.6%) were girls and 11 patients (31.4%) were boys. In the non‐HTN group, 32 patients (78.0%) were female and 9 patients (21.9%) were male. The difference in sex distribution between the two groups was not statistically significant (*p* value = 0.350).

Table 3 provides a summary of the laboratory results, comparing patients with normal blood pressure to those with HTN. Significant differences between the groups were observed in several parameters: Hgb and HCT levels at both the first and last visits, PMN cell count at the last visit, C3 at the first visit, Cr and BUN levels at the last visit, Na levels at the first visit, and Ca levels at both the first and last visits.

**TABLE 3:** Min, max, mean, and SD of laboratory assessments in HTN vs. non‐HTN groups.

Variable			Number	Mean	Standard deviation	Standard error	Independent samples test
Hgb	First visit	Normal BP	41	11.402	2.3991	0.3747	0.014^∗∗^
		HTN	35	10.054	2.2159	0.3746	
	Last visit	Normal BP	41	11.920	2.2657	0.3539	0.037^∗∗^
		HTN	35	10.820	2.2409	0.3788	

HCT	First visit	Normal BP	40	34.490	7.3038	1.1548	0.019^∗∗^
		HTN	33	30.639	6.2021	1.0796	
	Last visit	Normal BP	40	36.355	6.4329	1.0171	0.016^∗∗^
		HTN	33	32.655	6.3225	1.1006	

PLT	First visit	Normal BP	41	257.29	134.077	20.939	0.487
		HTN	34	234.71	145.239	24.908	
	Last visit	Normal BP	41	274.95	136.540	21.324	0.853
		HTN	34	269.00	139.551	23.933	

WBC	First visit	Normal BP	31	8.4717	4.68791	0.74122	0.341
		HTN	40	7.4742	3.85755	0.69284	
	Last visit	Normal BP	40	9.2590	3.95924	0.62601	0.208
		HTN	31	11.1848	11.1848	8.46735	

LYM percentage	First visit	Normal BP	40	31.06	15.7170	2.4851	0.705
		HTN	29	29.60	15.8437	2.9421	
	Last visit	Normal BP	40	27.343	15.6166	2.4692	0.116
		HTN	29	21.466	14.3991	2.6738	

PMN percentage	First visit	Normal BP	40	58.965	19.2907	3.0501	0.594
		HTN	29	61.338	16.4095	3.0472	
	Last visit	Normal BP	40	60.928	19.5874	3.0970	0.037^∗∗^
		HTN	30	70.293	16.2332	2.9638	

Chol		Normal BP	10	146.70	57.585	18.210	0.228
		HTN	21	180.71	77.438	16.898	

TG		Normal BP	10	182.60	72.984	23.080	0.209
		HTN	22	241.95	136.904	29.188	

ANA	First visit	Normal BP	12	40.608	80.4702	23.2297	0.263
		HTN	9	8.767	20.0473	6.6824	
	Last visit	Normal BP	12	40.908	80.3209	23.1867	0.258
		HTN	9	8.811	20.0301	6.6767	

C3	First visit	Normal BP	36	90.61	53.193	8.866	0.075
		HTN	31	66.55	55.350	9.941	
	Last visit	Normal BP	36	99.89	46.057	7.676	0.346
		HTN	31	87.94	57.053	10.247	

C4	First visit	Normal BP	36	16.478	13.4223	2.2370	0.447
		HTN	31	13.713	16.1640	2.9031	
	Last visit	Normal BP	36	19.367	14.2149	2.3691	0.987
		HTN	31	19.310	14.8248	2.6626	

ESR	First visit	Normal BP	39	48.74	42.645	6.829	0.827
		HTN	35	50.83	38.677	6.538	
	Last visit	Normal BP	38	37.03	31.806	5.160	0.826
		HTN	35	34.06	26.032	4.400	

CRP	First visit	Normal BP	38	8.229	14.1558	2.2964	0.295
		HTN	34	12.494	19.9009	3.4130	
	Last visit	Normal BP	37	5.646	11.8984	1.9561	0.396
		HTN	34	8.259	12.4360	2.1328	

Cr	First visit	Normal BP	40	0.825	0.6279	0.0993	0.135
		HTN	35	1.320	1.9602	0.3313	
	Last visit	Normal BP	40	0.660	0.1751	0.0277	0.015^∗∗^
		HTN	35	1.403	1.8701	0.3161	

BUN	First visit	Normal BP	40	20.200	37.0773	5.8624	0.638
		HTN	35	23.726	25.4625	4.3039	
	Last visit	Normal BP	40	13.03	7.804	1.234	0.000^∗∗^
		HTN	35	32.31	32.228	5.447	

Na	First visit	Normal BP	32	140.47	6.133	1.084	0.021^∗∗^
		HTN	34	137.12	5.330	0.914	
	Last visit	Normal BP	32	138.59	2.626	0.464	0.829
		HTN	34	137.76	3.955	0.678	

K	First visit	Normal BP	32	4.212	0.5552	0.0981	0.457
		HTN	34	4.315	0.5528	0.0948	
	Last visit	Normal BP	32	4.197	0.6219	0.1099	0.386
		HTN	34	4.329	0.6108	0.1047	

Ph	First visit	Normal BP	20	4.310	1.1011	0.2462	0.940
		HTN	29	4.338	1.3694	0.2543	
	Last visit	Normal BP	20	4.185	1.1753	0.2628	0.147
		HTN	29	4.862	1.8005	0.3343	

Ca	First visit	Normal BP	25	8.940	0.8031	0.1606	0.005^∗∗^
		HTN	31	8.303	0.8010	0.1439	
	Last visit	Normal BP	25	9.044	0.7372	0.1474	0.014^∗∗^
		HTN	31	8.471	0.9173	0.1648	

^∗∗^ *p*‐values under 0.05 are considered significant.

The mean pH‐VBG at the first visit was 7.38 (±0.07) and at the last visit was 7.33 (±0.20). For the first visit, PO2‐VBG, PCO2‐VBG, and the mean bicarbonate level of VBG were 62.82 (±41.08), 31.38 (±7.24), and 18.29 (±4.86), respectively. For the last visit, PO2‐VBG, PCO2‐VBG, and bicarbonate of VBG were 62.06 (±35.05), 34.29 (±9.82), and 18.80 (±5.44), respectively.

To identify independent factors associated with hypertension, a multivariable binary logistic regression analysis (Table 4) was performed including age at disease onset, sex, and lupus nephritis. Five cases with missing data were excluded, leaving 71 patients in the final model. The overall model was statistically significant (Omnibus test χ²=23.54, P‐value<0.001) and demonstrated good calibration (Hosmer–Lemeshow test, P‐value=0.759). The model explained 37.6% of the variance in hypertension (Nagelkerke R²=0.376) and correctly classified 77.5% of cases. In the multivariable analysis, lupus nephritis remained independently associated with hypertension. Patients with nephritis had significantly higher odds of hypertension compared with those without nephritis (adjusted OR=14.7, 95% CI: 4.2–52.6, p‐value<0.001). Neither age at onset (adjusted OR=0.95, 95% CI: 0.82–1.11, P‐value=0.530) nor sex (female vs. male: adjusted OR=0.89, 95% CI: 0.25–3.11, P‐value=0.852) was significantly associated with hypertension.

**TABLE 4:** Multivariable logistic regression analysis of factors associated with hypertension.

Variable	Adjusted OR	95% CI	*p* value
Age	0.95	0.82–1.11	0.530
Sex (female vs male)	0.89	0.25–3.11	0.852
Nephritis (yes vs no)	14.7	4.2–52.6	< 0.001

## 4. Discussion

This historical study evaluated clinical and laboratory characteristics, renal involvement, and HTN in children with systemic lupus erythematosus treated at the Children’s Medical Center from 2020 to 2022. Our findings provide valuable insights into the prevalence of HTN in cSLE patients and its association with various clinical and laboratory parameters.

HTN was present in 46.1% of the cSLE patients in our study. Overall, this prevalence aligns with previous studies indicating that HTN is a common comorbidity in adult and pediatric SLE patients, potentially due to renal involvement [13, 14]. In a study by Aydin et al., it was reported that HTN and prehypertension were present in 29% and 23% of cSLE patients at the first visit, but the difference in prevalence was not significant over time [7]. The slightly higher prevalence reported in our study could be explained by the fact that our hospital is a referral center in our country, where most severe cases are referred to.

Significant differences were observed between the HTN and non‐HTN groups in several laboratory parameters, including Hgb, HCT, PMN cell count, complement C3, Cr, BUN, sodium, and calcium levels. The significant differences in Cr and BUN levels, the markers of kidney function, particularly highlight the role of renal involvement in the pathogenesis of HTN in SLE patients [9]. Moreover, decreased complement levels, such as C3, may reflect active disease with a higher likelihood of renal flare‐ups, which could further contribute to HTN development [15].

Our study highlights clear gender predominance in participants, as most of the patients were female, constituting 73.7% of the total sample. As in our study, female predominance has also been reported in previous studies [16, 17]. Data analysis of the prevalence of HTN within these gender groups revealed HTN in 42.9% of the female patients (24 out of 56) and 55% of the male patients (11 out of 20). In this study, the difference in sex distribution between the two groups of HTN and non‐HTN was not statistically significant. In contrast, a cohort of 1214 adult SLE patients demonstrated that male patients had a significantly higher rate of HTN [18], which was in line with other studies [19, 20]. Our study also showed no statistically significant association between patients’ age and HTN. So, age did not appear to be an important risk factor for HTN in our study population.

LN was present in 59.2% of our patients. The kidney involvement in LN is one of the most severe complications in SLE. This condition is more prevalent and severe in children with greater associated morbidity and mortality [21, 22].

Some limitations could be considered for this study. The historical design and reliance on medical records may introduce bias, increase missing data, and limit the generalizability of our results. Systemic Lupus Erythematosus Disease Activity Index (SLEDAI) and Systemic Lupus International Collaborating Clinics/American College of Rheumatology Damage Index (SLICC/ACR Damage Index, SDI) scores were not consistently documented in the available records and thus could not be reliably extracted for analysis. Furthermore, the relatively small sample size could affect the statistical power to detect certain associations. Prospective studies with larger cohorts are needed to validate these findings and further explore the relationships between HTN, renal involvement, and overall disease activity in cSLE.

## 5. Conclusion

Our study provides valuable insights into the prevalence of HTN and its association with renal involvement in children with cSLE. Laboratory changes associated with increased disease activity, including lower C3 levels, lower Hbg, and higher BUN and Cr were reported in cSLE patients with HTN. The study findings have important implications for the management of HTN in cSLE and underscore the importance of regular monitoring and a tailored approach to manage this complex, multisystem disease to improve patient outcomes and reduce long‐term damage.

## Author Contributions

Fatemeh Tahghighi supervised the study from rheumatologic point of view, and Behnaz Bazargani, Arash Abbasi, Daryoush Fahimi, Fahimeh Askarian, Mastaneh Moghtaderi provided the patients’ information for this study. Noosha Samieefar critically revised the manuscript and consulted the methodology. All authors discussed the results and contributed to the data analysis and final manuscript.

## Funding

The authors have nothing to report.

## Disclosure

This study was a thesis project done by Mohamed Husein Aldokhi.

## Ethics Statement

The study was a historical study, so informed consent is not applicable. Ethical approval for this study was obtained from the Ethics Committee of Tehran University of Medical Sciences, allowing access to and extraction of patient data from hospital files and records (Batch Number: IR.TUMS.CHMC.REC.1402.05).

## Conflicts of Interest

The author declares no conflicts of interest.

## Data Availability

The data supporting this study’s findings are available from the corresponding author upon reasonable request.
